# Soluble Immune Complexes Shift the TLR-Induced Cytokine Production of Distinct Polarized Human Macrophage Subsets towards IL-10

**DOI:** 10.1371/journal.pone.0035994

**Published:** 2012-04-26

**Authors:** Carmen A. Ambarus, Kim C. M. Santegoets, Lenny van Bon, Mark H. Wenink, Paul P. Tak, Timothy R. D. J. Radstake, Dominique L. P. Baeten

**Affiliations:** 1 Department of Clinical Immunology and Rheumatology, Academic Medical Center/University of Amsterdam, Amsterdam, The Netherlands; 2 Department of Rheumatology, Radboud University Nijmegen Medical Center, Nijmegen, The Netherlands; National Council of Sciences (CONICET), Argentina

## Abstract

**Background:**

Costimulation of murine macrophages with immune complexes (ICs) and TLR ligands leads to alternative activation. Studies on human myeloid cells, however, indicate that ICs induce an increased pro-inflammatory cytokine production. This study aimed to clarify the effect of ICs on the pro- versus anti-inflammatory profile of human polarized macrophages.

**Materials and Methods:**

Monocytes isolated from peripheral blood of healthy donors were polarized for four days with IFN-γ, IL-4, IL-10, GM-CSF, M-CSF, or LPS, in the presence or absence of heat aggregated gamma-globulins (HAGGs). Phenotypic polarization markers were measured by flow cytometry. Polarized macrophages were stimulated with HAGGs or immobilized IgG alone or in combination with TLR ligands. TNF, IL-6, IL-10, IL-12, and IL-23 were measured by Luminex and/or RT-qPCR.

**Results:**

HAGGs did not modulate the phenotypic polarization and the cytokine production of macrophages. However, HAGGs significantly altered the TLR-induced cytokine production of all polarized macrophage subsets, with the exception of MΦ_IL-4_. In particular, HAGGs consistently enhanced the TLR-induced IL-10 production in both classically and alternatively polarized macrophages (M1 and M2). The effect of HAGGs on TNF and IL-6 production was less pronounced and depended on the polarization status, while IL-23p19 and IL-12p35 expression was not affected. In contrast with HAGGs, immobilized IgG induced a strong upregulation of not only IL-10, but also TNF and IL-6.

**Conclusion:**

HAGGs alone do not alter the phenotype and cytokine production of *in vitro* polarized human macrophages. In combination with TLR-ligands, however, HAGGs but not immobilized IgG shift the cytokine production of distinct macrophage subsets toward IL-10.

## Introduction

Macrophages play an important role in a wide variety of physiological and pathological processes including host defence, acute and chronic inflammation, and tissue homeostasis and remodelling. These pleiotropic cells can scavenge debris, sense microbial dangers signals, process and present antigens, and produce an array of pro- and anti-inflammatory mediators. Macrophage function, including the production of key cytokines such as TNF and IL-10, is not only determined by their activation but also by previous exposure to cytokines, growth factors, and other mediators during their differentiation from monocyte to macrophage. This so-called polarization process was originally proposed to distinguish classically activated macrophages (M1), which drive pro-inflammatory responses, from alternatively activated macrophages (M2), which steer immunoregulation and/or tissue remodelling [1]–[4]. Subsequent studies with mice and, to a lesser extent, human myeloid cells have lead to several more complex polarization models [5]–[7]. Using here the nomenclature proposed by Mantovani et al [5], the best characterized subsets are M1, M2a, and M2c, which are induced by IFN-γ, IL-4, or IL-10, respectively. Functional differences are accompanied by distinct phenotypic profiles, and we recently validated in vitro a number of specific phenotypic markers for each of these three macrophage subsets [8].

Of particular interest in the model proposed by Mantovani [5], are the so-called M2b macrophages, which result from polarization with ICs in combination with TLR ligands, such as LPS. Original studies showed that stimulation of mouse macrophages with ICs resulted in enhanced production of IL-10 and prostaglandins, especially PGE2 [9], while IL-6, IL-1, and TNF levels were not affected [10]–[12]. Polarization of mouse bone-marrow derived macrophages (BMDMs) with IFN-γ, followed by stimulation with ICs and LPS resulted also in an increased IL-10 production, which led to the conclusion that ICs modulate the macrophage cytokine production profile towards alternative activation, in a similar fashion as IL-10, TGF-β, or glucocorticoids [5], [7], [13]–[15].

Although this model has been confirmed by several studies, two important aspects remain incompletely understood. Firstly, it is unclear whether ICs induce macrophage polarization to a distinct subset or rather modulate the function of polarized macrophages. The previously mentioned experiments using IFN-γ polarized BMDMs could suggest namely either that M1 polarization can be reversed by ICs, or that ICs modulate the function of macrophages irrespective of their polarization status. Secondly, most of these experiments were performed in mice and only few studies analyzed the effects of ICs on human myeloid cells. In human monocytes, cross-linking of FcγRs decreased IL-12 and increased IL-1ra, IL-10, and PGE2 production, which is in agreement with the M2 profile in mice [16], [17]. The increased IL-10 production was not only observed after monocyte stimulation with artificial ICs, but also with ICs from SLE sera [18]. At the same time, however, the production of pro-inflammatory factors such as TNF, GM-CSF, IL-6, IL-8, and IL-1β by monocytes was also increased by FcγR cross-linking [19]–[24]. This was not only observed in human monocytes, since we demonstrated previously that costimulation of human monocyte-derived DCs with ICs and TLR ligands leads to increased production of TNF and IL-6 [25]. Similarly, stimulation of M-CSF polarized human macrophages (MΦ_M-CSF_) with immobilized HAGGs (iHAGGs) or ACPA-containing ICs induced higher TNF production [26], [27].

In order to clarify the effect of ICs on human macrophages and to assess whether the existing discrepancies in the literature are due to interspecies differences or to specific polarization conditions, we systematically studied the effect of HAGGs in the presence or absence of TLR stimuli on the phenotype and cytokine production of human polarized macrophages.

## Materials and Methods

### Ethics statement

This study was conducted with the approval of the Medical Ethical Committee of the Academic Medical Center/University of Amsterdam and all blood donors gave their written informed consent.

### Monocyte isolation and *in vitro* polarization

Monocyte isolation and in vitro polarization were performed as previously described [8]. Briefly, monocytes from peripheral blood of healthy volunteers were isolated by gradient centrifugation with Lymphoprep (Axis-Shield PoPAS, Oslo, Norway) and, subsequently, Percoll gradient separation (GE Healthcare, Uppsala, Sweden). Monocytes were cultured at a concentration of 0.5×10^6^/ml in Iscove's Modified Dulbecco's Medium (Invitrogen, Breda, The Netherlands) supplemented with 10% fetal calf serum (FCS) (PAA Laboratories, Cölbe, Germany) in 6 well culture plates (Corning Incorporated, New York, NY, USA). Cells were cultured in medium alone or polarized with human recombinant IFN-γ (50 ng/ml; R&D Systems, Abingdon, UK), IL-4 (40 ng/ml; Miltenyi Biotec, Bergisch Gladbach, Germany), IL-10 (50 ng/ml; R&D Systems), GM-CSF (50 ng/ml, R&D Systems), or M-CSF (50 ng/ml, R&D Systems) for 4 days. Polarization with LPS (100 ng/ml, E. coli 0111:B4; Sigma Aldrich, Zwijndrecht, The Netherlands) was additionally used for the phenotypic experiments. Human HAGGs (50 µg/ml), prepared as previously described [28], were added to each polarizing condition.

### Flow cytometric analysis

Cultured macrophages were recovered by scraping of the plate. Surface marker expression was analyzed by flow cytometry (BD FACS Calibur Flow Cytometer, Erembodegem, Belgium) using fluorochrome-labeled monoclonal antibodies against CD14 (clone 61D3, eBioscience, San Diego, CA), CD16 (clone DJ130c, AbD Serotec, Düsseldorf, Germany), CD32 (clone AT10, abcam, Cambridge, UK), CD64 (clone 10.1, BioLegend, Uithoorn, The Netherlands), CD80 (clone L307.4, BD Pharmingen, Breda, Nederland), CD163 (clone GHI/61, BD Pharmingen), CD200R (clone OX108, AbD Serotec), TLR2 (clone T2.5, abcam) and TLR4 (clone 76B357.1, abcam). Equivalent concentrations of matched isotype controls were included in all experiments. Before staining, Fc receptors were blocked with 10% human serum (Lonza, Cologne, Germany). Data were analyzed with Flow Jo Flow Cytometry Analysis software (Tree Star, Ashland, OR) after gating on the myeloid population in the FSC/SSC window. Values were expressed as the ratio of the geometric mean fluorescence intensity (gMFI) of the marker of interest over the gMFI of the isotype control.

### Cytokine production

To assess the cytokine production of polarized macrophages, the in vitro polarized cells were harvested and extensively washed at day 4. Macrophages were subsequently activated with HAGGs (50 µg/ml) and/or the following TLR ligands: LPS (100 ng/ml; Sigma Aldrich), Pam3CSK4 (5 µg/ml; EMC microcollections, Tübingen, Germany), or R848 (2 µg/ml; InvivoGen, San Diego, CA). In order to study the effect of immobilized IgG, macrophages were also cultured on IgG coated plates (96-well plate, Corning). The supernatant of these cultures was recovered after 20 hours of stimulation and analyzed using commercially available Luminex kits (Bio-Rad Laboratories, Hercules, CA) according to the manufacturer's instructions. Cytokine levels of TNF, IL-6, and IL-10 were measured and analyzed with the Bio-Plex system (Bio-Rad). The sensitivity of the cytokine assay was <5 pg/ml for all measured cytokines.

### Quantitative real-time PCR

Total RNA was isolated from in vitro polarized macrophages using GenElute™ Mammalian Total RNA Miniprep Kit (Sigma Aldrich) and reverse transcribed using RevertAid™ H Minus First Strand cDNA Synthesis Kit (Fermentas, St. Leon-Rot, Germany). RNA concentration was determined with the Nanodrop (Nanodrop Technologies, Wilmington, DE). Quantitative real-time PCR was performed using StepOnePlus™ Real-Time PCR System (Applied Biosystems, Foster City, CA). Each 12 µl reaction was performed in a 96-well format with 5 ng of cDNA, 10 µl of SYBR green PCR Master Mix (Applied Biosystems) and a concentration of 50 nmol of each primer. The primers comprised IL-10 (forward 5′-GATGCCTTCAGCAGAGTGAA, reverse 5′-CCCAGGTAACCCTTAAAGTCC), IL-23p19 (forward 5′-TTCTCTGCTCCCTGATAGCC, reverse 5′-CCTCAGGCTGCAGGAGTT), and IL-12p35 (forward 5′- ACCAGGTGGAGTTCAAGACC, reverse 5′-TGGCACAGTCTCACTGTTGA), respectively. All reactions were performed in duplicate. The mRNA expression levels were normalized to those of the human housekeeping gene glyceraldehyde 3-phosphate dehydrogenase (GAPDH). Oligonucleotide primers were designed using the online tool for Real-time PCR (TaqMan) Primer Design (Genscript) and obtained from Invitrogen.

### Statistics

Statistical analysis was performed using Prism software (GraphPad, La Jolla, CA). Data were expressed as mean ± SEM. ANOVA followed by Bonferroni post test were used for comparisons between more than 2 groups. For comparisons between 2 groups (stimulation with and without HAGGs) we used the Wilcoxon matched pair test. A *P* value of less than 0.05 was considered to be statistically significant.

## Results

### Soluble ICs do not alter phenotypic polarization of human macrophages

**Figure 1:**
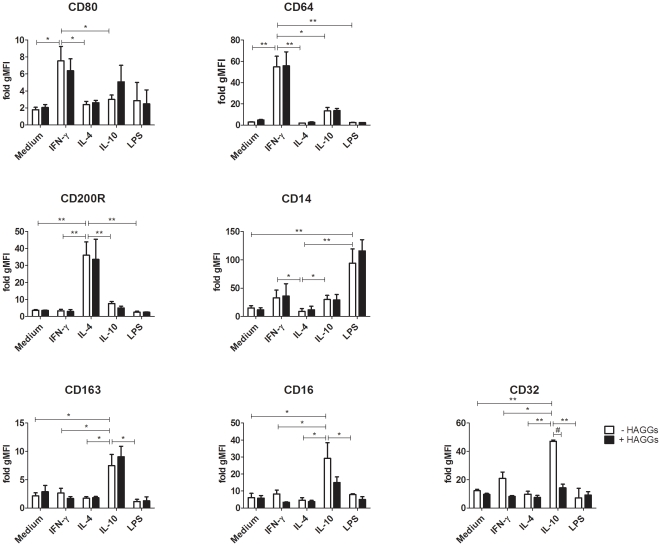
Expression of phenotypic markers on in vitro polarized human macrophages. Healthy peripheral blood monocytes were cultured for 4 days in medium or in medium supplemented with IFN-γ, IL-4, IL-10, or LPS in the absence (white bars) or presence (black bars) of HAGGs. Expression of MΦ_IFN-γ_ markers CD80 and CD64, MΦ_IL-4_ markers CD200R and CD14, and MΦ_IL-10_ markers CD163, CD16 and CD32 was measured by flow cytometry. Bars represent the mean ± SEM of 4 independent experiments. *p<0.05, **p<0.01, #p<0.01.

Several reports indicate that stimulation of murine macrophages with ICs in combination with TLR ligands induces alternative macrophage polarization [7], [10], [12]–[14], [29]. Therefore, our first aim was to investigate whether HAGGs had a similar polarizing effect on human macrophages. We tested whether the expression of phenotypic markers for specific polarized macrophage subsets was altered by HAGGs alone, in combination with LPS, or in combination with IFN-γ, IL-4, or IL-10 as major polarizing cytokines [5], [7]. IFN-γ polarization resulted in upregulation of CD80 (p<0.05 compared to medium, IL-4 and IL-10) and CD64 (p<0.05 compared to IL-10, and p<0.01 compared to medium, IL-4 and LPS), IL-4 upregulated CD200R (p<0.01 for all comparisons) and downregulated CD14 (p<0.05 compared to IFN-γ and IL-10, and p<0.01 compared to LPS), while IL-10 induced a higher expression of CD163 (p<0.05 for all comparisons), CD16 (p<0.05 for all comparisons), and CD32 (p<0.05 compared to IFN-γ, and p<0.01 compared to medium, IL-4 and LPS), respectively [8] (Figure 1). LPS used as polarizing stimulus strongly increased the expression of CD14 (p<0.01), as described previously [30]. Stimulation with HAGGs alone or in combination with LPS did not lead to upregulation of specific phenotypic markers, in particular those associated with IL-4 and IL-10 polarization (Figure 1). Additionally, HAGGs did not influence the phenotypic effects of IFN-γ, IL-4, or IL-10, with the exception of CD32 downregulation on MΦ_IL-10_ (p<0.01) (Figure 1). Taken together, these experiments did not show any polarizing effect of HAGGs on the phenotype of human macrophage subsets.

### Macrophage polarization determines the TLR-induced production of pro- and anti-inflammatory cytokines

**Figure 2:**
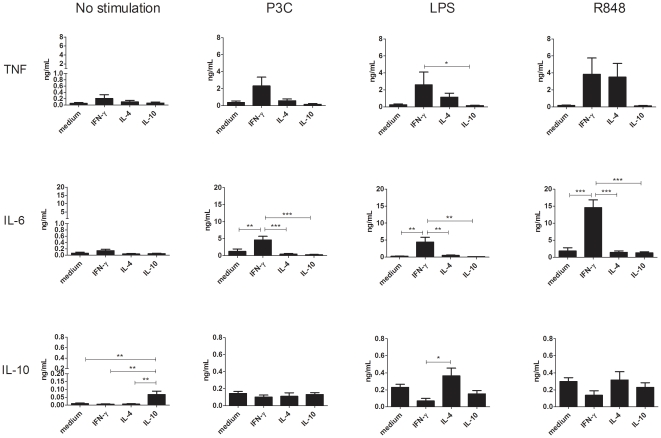
TLR-induced production of TNF, IL-6, and IL-10 by MΦ_IFN-γ_, MΦ_IL-4_, and MΦ_IL-10_. Healthy peripheral blood monocytes were cultured for 4 days in medium or in medium supplemented with IFN-γ, IL-4, or IL-10. Polarized macrophages were not stimulated, or stimulated for 20 hours with P3C, LPS, or R848. Bars represent the mean ± SEM of 6 independent experiments. *p<0.05, **p<0.01, ***p<0.001.

Numerous reports indicate that, besides phenotype, a main difference between distinct polarized macrophage subsets is the production of key cytokines such as TNF, IL-6, IL-10, IL-12 and IL-23 upon activation [5], [7], [13], [15], [31]–[33]. Before evaluating the effect of HAGGs on the cytokine production of polarized human macrophages, we first determined the cytokine production profile of MΦ_IFN-γ_, MΦ_IL-4_, and MΦ_IL-10_ upon TLR stimulation. As depicted in Figure 2, the production of TNF, IL-6 and IL-10 was very low for all macrophage subsets in the absence of TLR stimulation. In these basal conditions, MΦ_IL-10_ produced significantly more IL-10 than all other macrophage subsets (p<0.01). Following TLR stimulation, MΦ_IFN-γ_ produced more IL-6 than all other macrophage subsets (p<0.001 for the P3C and R848 stimulations, and p<0.01 for the LPS stimulation). There was a similar albeit less pronounced trend for TNF (p<0.05 versus MΦ_IL-10_ for the LPS stimulation), which was partially due to the fact that MΦ_IL-4_ also produced more TNF, especially after LPS and R848 stimulation. IL-10 levels were similar in all polarized subsets, with the exception of a higher production by MΦ_IL-4_ versus MΦ_IFN-γ_ after LPS stimulation (p<0.05). Despite the fact that some comparisons did not reach statistical significance, these findings confirm previous reports of a pro-inflammatory profile of MΦ_IFN-γ_ versus a more neutral to anti-inflammatory profile for MΦ_IL-4_ and MΦ_IL-10_ upon TLR stimulation [5], [7], [13], [15].

### Soluble ICs enhance the TLR-induced IL-10 production by polarized macrophages

**Figure 3:**
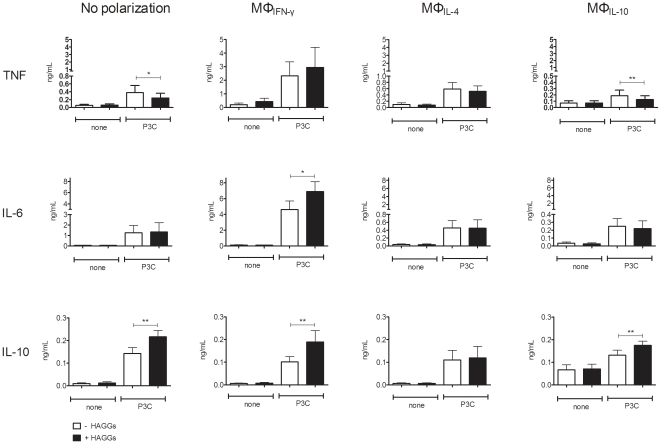
TNF, IL-6, and IL-10 production by MΦ_IFN-γ_, MΦ_IL-4_, and MΦ_IL-10_ after stimulation with HAGGs and P3C. Healthy peripheral blood monocytes were cultured for 4 days in medium or in medium supplemented with IFN-γ, IL-4, or IL-10. Polarized macrophages were not stimulated, or stimulated for 20 hours with P3C, in the presence or absence of HAGGs. Bars represent the mean ± SEM of 9 independent experiments. *p<0.05, **p<0.01.

**Figure 4:**
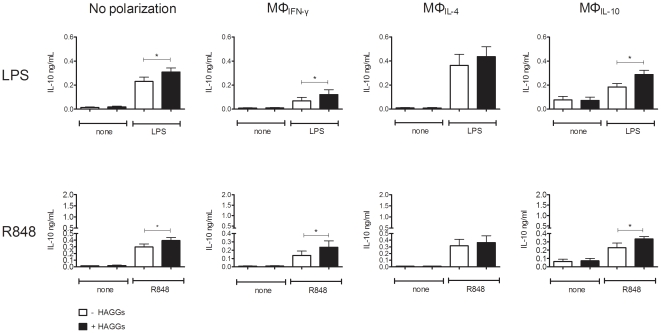
IL-10 production by MΦ_IFN-γ_, MΦ_IL-4_, and MΦ_IL-10_ after stimulation with HAGGs and LPS, or R848. Healthy peripheral blood monocytes were cultured for 4 days in medium or in medium supplemented with IFN-γ, IL-4, or IL-10. Polarized macrophages were not stimulated, or stimulated for 20 hours with LPS, or R848, in the presence or absence of HAGGs. Bars represent the mean ± SEM of 6 independent experiments. *p<0.05.

Since ICs were described to induce an immunoregulatory function in mouse macrophages [10], [18], [29], [34], [35], we next investigated whether HAGGs alter the pro- versus anti-inflammatory cytokine production of differentially polarized human macrophages. In the absence of TLR stimulation, HAGGs did not alter the low basal production of TNF, IL-6, and IL-10 by any of the macrophage subsets (Figure 3). Using P3C as prototypical TLR stimulation, costimulation with HAGGs had variable effects on the production of pro-inflammatory cytokines, with a decrease in TNF production by unpolarized macrophages and MΦ_IL-10_, and an increased IL-6 production by MΦ_IFN-γ_ (p<0.05 compared to P3C alone for all 3 comparisons) (Figure 3). In contrast, HAGGs consistently enhanced IL-10 production by all macrophage subsets, with the exception of MΦ_IL-4_ (p<0.01 for all comparisons) (Figure 3). In particular, HAGGs almost doubled the IL-10 secretion by MΦ_IFN-γ_. These data were confirmed by repeating the experiments with other TLR stimuli. HAGGs together with LPS or R848 induced a modest and variable effect on TNF and IL-6 production (data not shown), but a consistent and significant increase in IL-10 production by all macrophage subsets, with the exception of MΦ_IL-4_ (p<0.05 compared to TLR stimulation without HAGGs) (Figure 4). Thus, costimulation of in vitro polarized human macrophages with HAGGs and TLR ligands resulted in an increased IL-10 production by all polarized macrophage subsets, excepting MΦ_IL-4_.

### Effect of soluble ICs on the IL-10 production by MΦ_M-CSF_ and MΦ_GM-CSF_

**Figure 5:**
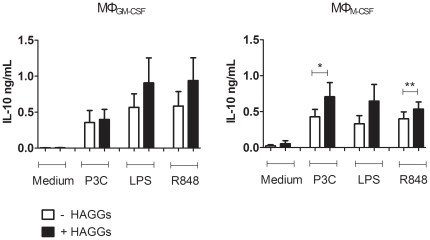
IL-10 production by MΦ_GM-CSF_ and MΦ_M-CSF_ after stimulation with HAGGs and TLR ligands. Healthy peripheral blood monocytes were cultured for 4 days in medium supplemented with GM-CSF or M-CSF. MΦ_GM-CSF_ and MΦ_M-CSF_ were not stimulated, or stimulated for 20 hours with P3C, LPS, or R848, in the presence or absence of HAGGs. Bars represent the mean ± SEM of 6 independent experiments. *p<0.05, **p<0.01.

Both phenotypic analyses and cytokine profiling indicated that GM-CSF polarized macrophages (MΦ_GM-CSF_) tend to resemble MΦ_IFN-γ_, whereas M-CSF mimics the effects of alternative polarization stimuli, in particular IL-10 [8], [32], [33], [36]. We therefore investigated whether stimulation with HAGGs also modulated the IL-10 production of MΦ_GM-CSF_ and MΦ_M-CSF_. HAGGs alone did not have any effect on the IL-10 production. However, after co-stimulation with TLR ligands, HAGGs induced an increased IL-10 production by MΦ_M-CSF_ but not MΦ_GM-CSF_ (p<0.05 for P3C, and p<0.01 for R848) (Figure 5). Concluding, MΦ_M-CSF_ showed a higher TLR-induced IL-10 production after stimulation with HAGGs.

### Increased LPS-induced IL-10 versus IL-23 and IL-12 expression after stimulation with soluble ICs

**Figure 6:**
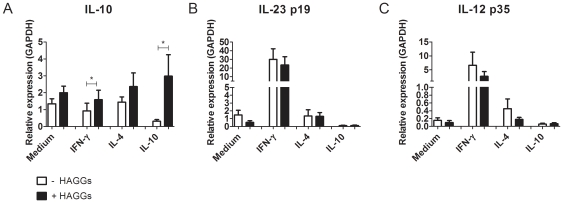
mRNA expression of IL-10, IL-23p19, and IL-12p35 after macrophage stimulation with HAGGs and LPS. Healthy peripheral blood monocytes were cultured for 4 days in medium or in medium supplemented with IFN-γ, IL-4, or IL-10. Polarized macrophages were stimulated for 7 hours with LPS, in the presence or absence of HAGGs. mRNA expression levels were measured by quantitative RT-PCR and were normalized to those of the human housekeeping gene GAPDH. Bars represent the mean ± SEM of 6 independent experiments. *p<0.05.

In order to confirm the increase in TLR-induced IL-10 production by polarized macrophages after stimulation with HAGGs, we measured the mRNA expression of IL-10 by each macrophage subset after 7 hours of stimulation with LPS, in the presence or absence of HAGGs. As shown in figure 6, HAGGs increased the expression of IL-10 in all macrophage subsets (p<0.05 for MΦ_IFN-γ_ and MΦ_IL-10_). As IL-23 and IL-12 protein levels were undetectable in the supernatants of the previous experiments, we investigated the effect of HAGGs on these cytokines at mRNA level. In contrast with IL-10, we did not observe any significant effect of HAGGs on IL-23p19 and IL-12p35 mRNA expression. Taken together, these data confirm the specific increase in IL-10 expression after costimulation with HAGGs and TLR ligands, with the strongest effects observed in MΦ_IFN-γ_ and MΦ_IL-10_.

### Immobilized IgG enhances the cytokine production of polarized macrophages without shifting the balance towards IL-10

**Figure 7:**
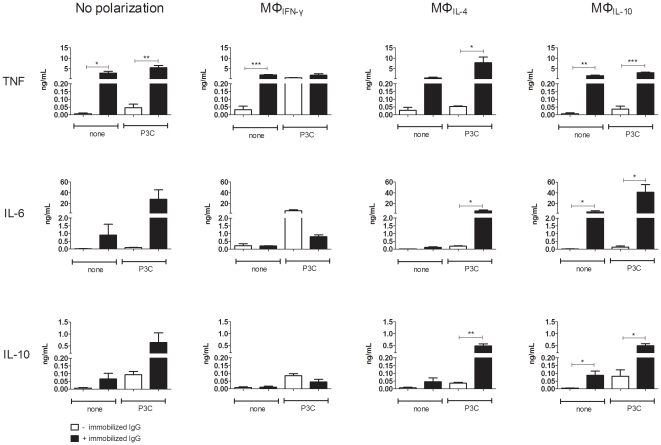
TNF, IL-6, and IL-10 production by MΦ_IFN-γ_, MΦ_IL-4_, and MΦ_IL-10_ after stimulation with immobilized IgG and P3C. Healthy peripheral blood monocytes were cultured for 4 days in medium or in medium supplemented with IFN-γ, IL-4, or IL-10. Polarized macrophages were not stimulated, or stimulated for 20 hours with P3C, in the presence or absence of immobilized IgG. Bars represent the mean ± SEM of 3 independent experiments. *p<0.05, **p<0.01, ***p<0.001.

As the consistent increase in IL-10 production by HAGGs in our experiments contrasted with previously published data on increased TNF production by human macrophages upon stimulation with iHAGGs or immobilized IgG [26], [27], [37], we next investigated the effect of immobilized IgG on the cytokine production of differentially polarized macrophages. In contrast with HAGGs, even in the absence of P3C stimulation, immobilized IgG increased the production of TNF (p<0.05 in unpolarized macrophages, p<0.01 in MΦ_IL-10_, and p<0.001 in MΦ_IFN-γ_), IL-6 (p<0.05 in MΦ_IL-10_) and IL-10 (p<0.05 in MΦ_IL-10_) (Figure 7). Similarly, immobilized IgG strongly augmented the TLR-induced production of TNF (p<0.05 in MΦ_IL-4_, p<0.01 in unpolarized macrophages, and p<0.001 in MΦ_IL-10_), IL-6 (p<0.05 in MΦ_IL-4_ and MΦ_IL-10_) and IL-10 (p<0.05 in MΦ_IL-10_, and p<0.01 in MΦ_IL-4_). Comparable results were obtained when using LPS or R848 as TLR stimuli (data not shown). These data indicate that immobilized IgG augments the spontaneous, as well as the TLR-induced cytokine production of polarized macrophages, without shifting the balance from pro-inflammatory cytokines to IL-10.

## Discussion

ICs in combination with TLR ligands have been described to increase the IL-10 production of mouse macrophages, leading to a distinct alternative macrophage activation type (M2b) [5]. ICs also stimulated the in vivo IL-10 production in different animal models, which induced immune regulation [34], [38]. In host defence, this mechanism could represent a physiological feed-back loop, contributing to the down-regulation of innate tissue inflammation at the moment that microorganisms are cleared by the adaptive immune system. The effect of ICs on human myeloid cells, however, is less clearly defined, as several reports indicate that they do not only increase the production of anti-inflammatory mediators such as IL-10 [16]–[18], but also strongly upregulate pro-inflammatory mediators [19]–[27]. This discrepancy may be related to differences between species, cell types (monocytes versus macrophages, or dendritic cells), or macrophage polarization conditions. In this study we show that HAGGs in the absence of TLR stimuli do not alter the phenotype and cytokine production of in vitro polarized human macrophage subsets. Costimulation with HAGGs and TLR ligands, however, resulted in markedly increased IL-10 production in most macrophage subsets, while the modulation of TNF and IL-6 production was more discrete and dependent on the macrophage polarization status. As previously shown in mouse macrophages [10], [12], [13], [29], costimulation with ICs and TLR ligands also augmented the IL-10 production of human MΦ_IFN-γ_, which are prototypical pro-inflammatory cells.

These findings raise the critical issue of the exact relationship between polarization, phenotype and function of human macrophages. The macrophage polarization models originally refer to the production of pro- versus anti-inflammatory mediators to define classically (M1) versus alternatively activated macrophages (M2) [1]–[3], [5]–[7]. Macrophage polarization is also associated with the expression of specific phenotypic markers [4], [8], [8], [39]–[41]. Our experiments confirm that polarization affects both the phenotype and the function of macrophages, as exemplified by the distinct cytokine production of MΦ_IFN-γ_, MΦ_IL-4_, and MΦ_IL-10_ upon TLR stimulation. Concerning the effect of ICs, we show that HAGGs can modulate the TLR-mediated cytokine production of human polarized macrophages, despite the lack of phenotypic alterations. The capacity of so-called ‘pro-inflammatory’ macrophages to produce significant amounts of IL-10 upon costimulation with HAGGs and TLR ligands illustrate the functional plasticity of these cells. Furthermore, our data indicate that the production of pro- versus anti-inflammatory mediators by macrophages is not only related to their polarization, but also to the type of activation and that, as a consequence, phenotypic polarization markers do not fully correlate to the macrophage function.

An important aspect in this context is the expression of FcγRs on polarized macrophage subsets. Studies using different IgG subtypes, specific FcγR blocking antibodies, or FcγR knockout animals demonstrated that the differential expression of the activating versus inhibitory FcγRs on immune cells influences their response to ICs [23], [28], [37], [42]–[45]. We previously confirmed that the high affinity FcγRI (CD64) was upregulated on MΦ_IFN-γ_, while the low affinity FcγRII (CD32) and FcγRIII (CD16) were specifically expressed by MΦ_IL-10_. Furthermore, the activating and inhibitory isoforms of CD32 (CD32a and CD32b, respectively) were differentially expressed on the 3 macrophage subsets, with a lower CD32a/CD32b ratio on MΦ_IL-4_ versus MΦ_IL-10_ and MΦ_IFN-γ_ [8]. A shifted FcγR balance towards FcγRIIb on IL-4 primed human monocytes in comparison to IFN-γ, TNF, and IL-10 primed monocytes was previously related to the failure of these cells to upregulate TNF after crosslinking by IgG [37]. Finally, also the expression of TLR2 and TLR4 was differentially modulated by polarization, with lower expression levels on MΦ_IL-4_ versus MΦ_IL-10_ and MΦ_IFN-γ_ (Figure S1). Although it is beyond the scope of this study to investigate the exact signal transduction in these conditions, the low expression of activating FcγRs, TLR2, and TLR4 on MΦ_IL-4_ could at least partially explain their poor responsiveness to HAGGs.

In an attempt to clarify the discrepancy between our findings and reports indicating increased TNF production by monocytes and macrophages upon stimulation with immobilized IgG [26], [27], [37], we used immobilized IgG in the same experimental conditions and observed a number of differences with soluble HAGGs. Firstly, immobilized IgG, but not HAGG was able to stimulate cytokine production by polarized macrophages in the absence of TLR co-stimulation. Secondly, whereas MΦ_IL-4_ appeared to be poorly responsive to HAGGs, all polarized macrophage subsets reacted strongly upon stimulation with immobilized IgG. Finally and most importantly, HAGGs consistently augmented IL-10 production, whereas immobilized IgG upregulated the production of TNF, IL-6 and IL-10. These data are consistent with previous reports in which prevention of IC phagocytosis by using immobilized IgG was shown to induce persistent ERK signaling and high TNF production by both monocytes and macrophages [26], [46], [47]. Furthermore, the TLR-induced IL-10 production by human monocytes was higher after immobilized IgG versus HAGG stimulation, and immobilized IgG was able to induce IL-10 production even in the absence of TLR ligands [48]. This data emphasize that, besides many other factors such as IgG isotype, monomeric versus polymeric IgG, antigen-antibody ratio, and natural versus artificial ICs, the capacity to internalize ICs is a key determinant of the functional outcome of IC stimulation of macrophages [16], [18]–[23], [42], [49].

The differential effects of soluble versus immobilized IgG do not only emphasize the functional plasticity of polarized macrophages, but may also be relevant for our understanding of specific pathological and therapeutic conditions. In autoimmune diseases, ICs are thought to contribute to chronic tissue inflammation when deposited in tissues, with as prototypical examples lupus nephritis and rheumatoid synovitis. In this immobilized form, ICs may promote mainly pro-inflammatory over anti-inflammatory responses by tissue macrophages. Moreover, IC binding to myeloid cells in these conditions was also reported to inhibit their responsiveness to IL-10 [50], [51]. In sharp contrast, intravenous administration of soluble immunoglobulins (IVIg) has been used for a long time in the treatment of diverse autoimmune diseases [52]–[55]. The immunosuppressive effect of macrophage FcγR ligation has been attributed to blocking of the activating FcγRs, stimulation of FcγRIIb, and increased anti- versus pro-inflammatory cytokine production [10], [12], [16], [18], [45], [56]. Our in vitro data thus support the hypothesis that soluble ICs shift the balance of pro- towards anti-inflammatory cytokine production. Interestingly, this effect may be important not only for the therapeutic efficacy of IVIg, but also of targeted biological therapies. It was indeed recently demonstrated that anti-TNF antibodies induce IL-10 producing macrophages in an Fc-dependent manner and that these immunoregulatory macrophages are involved in mucosal healing in inflammatory bowel disease [56], [57]. These studies reveal that monoclonal antibody therapy can drive anti-inflammatory responses by Fc region-dependent and target-independent modulation of macrophage function.

In conclusion, we showed here that distinct polarized macrophage subsets retain an important functional plasticity despite maintenance of their specific phenotype. In particular, we demonstrated that soluble ICs, but not immobilized IgG shifted the balance of human macrophage cytokine production towards IL-10. These findings raise the possibility of therapeutic modulation of macrophage function in the context of chronic tissue inflammation.

## Supporting Information

Figure S1 **Expression of TLR2 and TLR4 on MΦ_IFN-γ_, MΦ_IL-4_, and MΦ_IL-10_.** Healthy peripheral blood monocytes were cultured for 4 days in medium or in medium supplemented with IFN-γ, IL-4, or IL-10. Expression TLR2 and TLR4 was measured by flow cytometry. Bars represent the mean ± SEM of 4 independent experiments. *p<0.05, **p<0.01
(TIF)
