# Supplementary figures and images for: Soluble Immune Complexes Shift the TLR-Induced Cytokine Production of Distinct Polarized Human Macrophage Subsets towards IL-10
Source: PLoS One. 2012 Apr 26;7(4):e35994. doi: 10.1371/journal.pone.0035994 (PMC3338562; doi:10.1371/journal.pone.0035994)

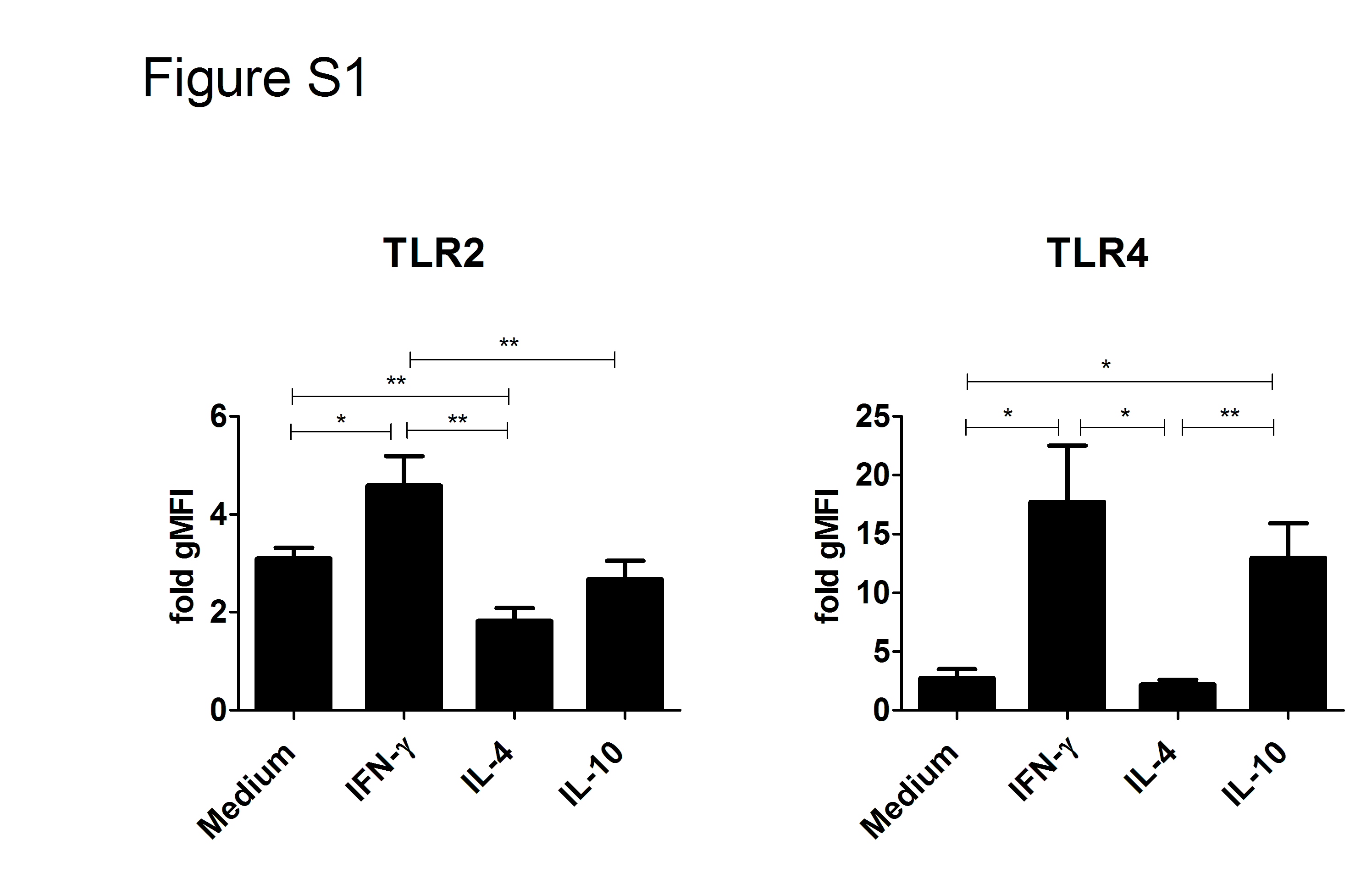

Supplement: Figure S1 — Expression of TLR2 and TLR4 on MΦIFN-γ, MΦIL-4, and MΦIL-10. Healthy peripheral blood monocytes were cultured for 4 days in medium or in medium supplemented with IFN-γ, IL-4, or IL-10. Expression TLR2 and TLR4 was measured by flow cytometry. Bars represent the mean ± SEM of 4 independent experiments. *p<0.05, **p<0.01 (TIF) [file pone.0035994.s001.tif]
